# Atomic Pt-Layer-Coated Au Peroxidase Nanozymes with Enhanced Activity for Ultrasensitive Colorimetric Immunoassay of Interleukin-12

**DOI:** 10.3390/bios15040239

**Published:** 2025-04-09

**Authors:** Han Zhang, Xiang Peng, Hao Song, Yongfeng Tan, Jianglian Xu, Qunfang Li, Zhuangqiang Gao

**Affiliations:** 1 School of Chemistry and Environmental Engineering, Hubei Minzu University, Enshi 445000, China; 17852602023@163.com; 2 Marshall Laboratory of Biomedical Engineering, Shenzhen Key Laboratory for Nano-Biosensing Technology, School of Biomedical Engineering, Shenzhen University Medical School, Shenzhen University, Shenzhen 518060, China; zora1951@163.com (X.P.); 15249939659@163.com (H.S.); tanyongfeng0@gmail.com (Y.T.); jianglianxu0916@gmail.com (J.X.)

**Keywords:** peroxidase nanozymes, (Au core)@(Pt atomic-layer-shell) nanoparticles, colorimetric enzyme-linked immunosorbent assay, interleukin-12, high sensitivity

## Abstract

Interleukin-12 (IL-12), a crucial biomarker for immune and inflammatory responses, plays a pivotal role in diagnosing and managing diverse pathological conditions. Although colorimetric enzyme-linked immunosorbent assays (CELISAs) have been extensively employed to detect IL-12 in biological samples, their sensitivity is inherently limited by the catalytic efficiency of enzyme labels, presenting substantial challenges in achieving ultrasensitive detection and enabling pre-symptomatic diagnosis of diseases. In this study, we address this limitation by developing a novel peroxidase nanozyme, featuring ultrathin Pt skins consisting of only ~4 atomic layers, coated on Au nanoparticles (denoted as Au@Pt_4L_NPs). These Au@Pt_4L_NPs exhibit remarkable catalytic performance, achieving a ~1063-fold enhancement in peroxidase-like activity compared to horseradish peroxidase (HRP), while minimizing Pt consumption, thereby improving Pt utilization efficiency and reducing costs. This advancement facilitates the construction of an ultrasensitive CELISA capable of detecting IL-12 at femtomolar concentrations. Using Au@Pt_4L_NPs as the signal labels, the developed CELISA demonstrates a quantitative detection range from 0.1 to 100 pg mL^−1^, with a limit of detection (LOD) as low as 0.084 pg mL^−1^ (1.1 fM), offering ~10 times greater sensitivity than the HRP-based CELISA. This study highlights the potential of Au@Pt_4L_NP nanozymes as advanced signal labels, opening new avenues for next-generation ultrasensitive bioassays.

## 1. Introduction

Interleukin-12 (IL-12) is a potent proinflammatory cytokine primarily produced by myeloid cells, including dendritic cells and macrophages, in response to infections. As a critical mediator in the immune system, IL-12 drives the differentiation of naive T cells into T helper 1 cells and stimulates the secretion of interferon-γ, which is essential for cellular immunity and defense against intracellular pathogens. Additionally, IL-12 is a central regulator of both innate and adaptive immune responses, influencing the activity of natural killer cells and supporting the function of cytotoxic T lymphocytes. Clinically, IL-12 has emerged as an important biomarker for immune-system activation and inflammation responses. Elevated IL-12 concentrations are linked to a wide range of diseases, including chronic infections, autoimmune conditions, inflammatory disorders, and cancer [1,2,3]. For example, high IL-12 concentrations are often observed in patients with autoimmune diseases, including rheumatoid arthritis, psoriasis, and inflammatory bowel diseases [4,5,6]. Additionally, IL-12 has been implicated in the pathogenesis of various cancers, where its dysregulation can contribute to tumor progression and metastasis [7]. The clinical significance of IL-12 extends beyond being a biomarker, as it also holds promise as a therapeutic target. Its role in modulating immune responses has led to its exploration as a potential agent in immunotherapy. By enhancing the activity of Th1 cells and NK cells, IL-12 can help boost the body’s natural ability to combat tumors and infections. Consequently, recombinant IL-12 has been investigated as an adjuvant in immunotherapy, with promising results in preclinical and clinical trials for certain inflammatory diseases [3]. Given its central role in immune regulation and disease pathogenesis, the sensitive detection of IL-12 in biological fluids, such as serum or plasma, is of great importance for the diagnosis, monitoring, and management of diseases. It aids in assessing immune system activity, evaluating the severity of inflammatory conditions, and tailoring personalized therapeutic strategies. Accurate and reliable methods for measuring IL-12 levels are crucial for advancing diagnostics and improving patient outcomes in various immunological and oncological scenarios.

To date, various immunoassay-based approaches have been developed for detecting IL-12, combining the high specificity of antigen–antibody interactions with the high sensitivity enabled by diverse signal generation mechanisms (e.g., colorimetry, luminescence, and electrochemistry) and advanced signal amplification strategies (e.g., nucleic acid amplification, tyramide signal amplification, and polymer–enzyme signal amplification) [8,9,10,11]. Within these immunoassay-based approaches, colorimetric enzyme-linked immunosorbent assays (CELISAs) are among the most commonly used approaches due to their simple detection principle, which amplifies antigen–antibody binding events through enzymatic reactions that generate visually detectable color signals. CELISAs are widely favored in clinical diagnostics for their straightforward operation, reliability, affordability, and compatibility with routine laboratory workflows, establishing them as the gold-standard method for IL-12 detection [12,13]. Despite these strengths, conventional CELISAs face inherent limitations in sensitivity, which constrain their ability to detect ultra-low levels of IL-12 [14]. Note that in healthy individuals, circulating IL-12 is typically present at ultra-low levels, often falling below the detection limits (LODs) of conventional CELISAs (LOD = 1.0~4.0 pg mL^−1^, as summarized in Table S1). Elevated IL-12 levels generally become detectable only during advanced or acute disease stages, frequently after clinical symptoms have manifested. This limitation highlights the urgent need for innovative assay technologies that can detect IL-12 at trace levels (~fg mL^−1^, ~fM) to enable early, pre-symptomatic diagnosis and improve therapeutic outcomes [15].

Because the signal generation in CELISAs relies primarily on the catalytic conversion of substrates by natural enzymes, such as horseradish peroxidase (HRP), to yield colorimetric outputs, the sensitivity of this technique is fundamentally constrained by the enzymes’ catalytic efficiency [16,17]. To overcome this limitation, an effective strategy involves replacing conventional enzymes with advanced catalysts exhibiting superior catalytic performance, thereby amplifying colorimetric signals and enhancing detection sensitivity [17]. Inorganic nanostructure-based peroxidase mimics (nanozymes) have recently gained attention as promising alternatives for advanced bioassay applications. By meticulously engineering parameters such as composition, morphology, and structural attributes, the catalytic characteristics of these peroxidase nanozymes are capable of being optimized to fulfill the demands of highly sensitive bio-assays [18,19,20,21,22]. The initial discovery of peroxidase-like activity in Fe_3_O_4_ nanoparticles, reported by Yan et al. in 2007, opened the door to exploring nanostructures with enzyme-mimetic properties [23]. Since then, a wide range of peroxidase nanozymes have been developed, including nanomaterials made of metal oxides, metal nitrides, metal selenides, metal sulfides, carbon, metal-organic frameworks, and noble metals [24,25,26,27,28,29,30,31,32,33]. Compared to natural enzymes, these nanozymes offer several notable advantages, including straightforward synthesis, greater stability, enhanced catalytic activity, and ease of storage, positioning them as ideal candidates for bioanalytical applications [18,19,20,21,22]. Among these, (Au core)@(Pt shell) nanoparticles (Au@PtNPs) have recently gained attention as highly promising peroxidase nanozymes, offering great potential for ultrasensitive colorimetric immunoassays [33,34,35,36,37,38]. Their advantages are multifaceted: (i) Au@PtNPs exhibit significantly higher catalytic activity compared to nanozymes composed of other materials, enabling higher sensitivity in immunoassays; (ii) they are synthesized with exceptional purity and uniformity, ensuring the high reproducibility of assay results; (iii) they show excellent stability in assay conditions and maintain functionality during reactions, thus enhancing assay robustness; and (iv) they are easily functionalized with a range of biomolecules via thiol-Pt chemistry, facilitating efficient antibody conjugation and flexible assay configurations. Despite these advantages, the widespread use of Au@PtNPs is hindered by the high consumption of Pt, a rare and costly element, which limits their large-scale application in ultrasensitive CELISAs [39,40,41]. This highlights the urgent need to design innovative Au@PtNP-based peroxidase nanozymes with improved mass-specific catalytic activity (catalytic activity per unit mass of Pt, s^−1^ mg^−1^_Pt_). By significantly boosting the catalytic efficiency while reducing Pt consumption, it would be possible to achieve the ultrasensitive detection of IL-12 in a cost-effective manner, paving the way for the practical adoption of these advanced nanozymes in diagnostic technologies.

**Scheme 1:**
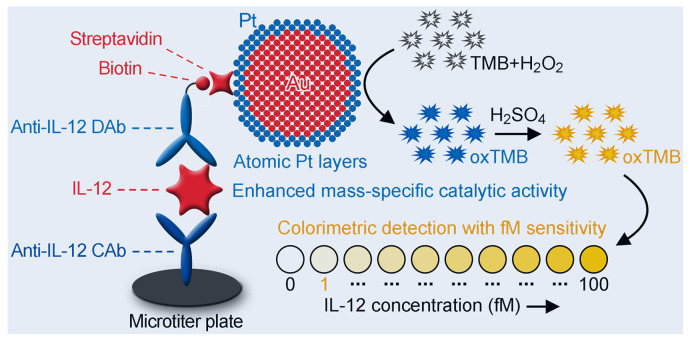
Schematic illustration showing the working principle of the ultrasensitive CELISA for the detection of IL-12 at femtomolar levels using Au@Pt_4L_NP-based peroxidase nanozymes as the signal labels.

In this study, we report the engineering of (Au core)@(Pt atomic-layer-shell) nanoparticles as highly efficient peroxidase nanozymes for developing an ultrasensitive CELISA platform capable of detecting IL-12 at femtomolar levels (Scheme 1). These nanoparticles are constructed by carefully controlling the reaction kinetics during synthesis, ensuring that the surface diffusion rate of Pt atoms significantly exceeds their deposition rate (*V*_dep._ << *V*_diff._) [42,43]. This precise kinetic regulation allows the uniform coating of Au nanoparticles (AuNPs) with ultrathin Pt shells consisting of only four atomic layers (denoted as Au@Pt_4L_NPs). This structural design maximizes catalytic performance while minimizing Pt consumption, making Au@Pt_4L_NPs particularly suitable for enhancing the sensitivity and cost-efficiency of CELISAs. To detect IL-12, the CELISA platform is designed with microplate wells immobilized with anti-IL-12 capture antibodies (CAbs). Biotinylated anti-IL-12 detection antibodies (biotin-anti-IL-12 DAbs) are used to recognize the target IL-12, and streptavidin (SA)-conjugated Au@Pt_4L_NPs (SA-Au@Pt_4L_NP conjugates) serve as the signal amplification probes. This setup facilitates the formation of a sandwich immunocomplex (anti-IL-12 CAb/IL-12/biotin-anti-IL-12 DAb/Au@Pt_4L_NP-SA) in the microplate wells. Upon introducing the substrates, 3,3′,5,5′-tetramethylbenzidine (TMB) and hydrogen peroxide (H_2_O_2_), the Au@Pt_4L_NPs catalyze the TMB and H_2_O_2_ substrates, generating an intense color change as the signal for ultrasensitive detection.

## 2. Materials and Methods

### 2.1. Preparation of ~40 nm AuNPs

Initially, ~15 nm AuNPs as seeds were synthesized via a modified version of the classic Frens’ method (refer to the Supporting Information for the experimental details) [44]. Then, ~40 nm AuNPs were synthesized via a modified seed-mediated growth approach, utilizing the ~15 nm AuNPs as seeds [45]. In brief, 36 mL of deionized (DI) water was combined with 4 mL of ~15 nm AuNPs in a 100 mL flask. The mixture was maintained at room temperature (~25 °C) under continuous magnetic stirring. Subsequently, a 20 mL precursor solution containing 0.04% (*w*/*v*) HAuCl_4_ and a 20 mL reductant solution containing 0.06% (*w*/*v*) L-ascorbic acid and 0.06% (*w*/*v*) sodium citrate were separately injected into the ~15 nm AuNPs suspension at a controlled rate of 200 µL/min using a syringe pump. Following the completion of the injection process, the reaction mixture was subjected to boiling with continuous stirring for 30 min. After cooling down, the produced ~40 nm AuNPs were stored at room temperature for future use. The concentration of the final ~40 nm AuNPs suspension was determined to be ~9.09 × 10^10^ particles/mL (~1.51 × 10^−10^ M).

### 2.2. Synthesis of Au@Pt_4L_NPs

The Au@Pt_4L_NPs were synthesized via a straightforward seed-mediated growth method, utilizing the above ~40 nm AuNPs as seeds [35,36]. In a standard synthesis, 8.0 mL of the above ~40 nm AuNPs, 3.622 mL of DI water, 378 µL of 1 mM K_2_PtCl_6_, and 4.0 mL of 1.0 mM L-ascorbic acid were sequentially introduced into a glass vial. The resulting mixture was then incubated in an oven set to 80 °C for 3 h. After the reaction, the produced Au@Pt_4L_NPs (with the Pt-to-Au molar ratio of 0.163) were then cooled and stored at room temperature for future use. The concentration of the final Au@Pt_4L_NPs suspension was determined to be ~4.55 × 10^10^ particles/mL (7.55 × 10^−11^ M). Syntheses of Au@PtNPs with Pt shells of different Pt-to-Au molar ratios followed similar procedures, with specific reagent adjustments outlined in Table S2.

### 2.3. Investigation of Peroxidase-like Catalytic Activity of Au@Pt_4L_NPs

The peroxidase-like catalytic activity of Au@Pt_4L_NPs was investigated by incubating Au@Pt_4L_NPs (final concentration: 3.78 × 10^−14^ M) in a substrate solution composed of a citrate–phosphate buffer (pH 4.0) containing 0.8 mM TMB and 7 M H_2_O_2_. The reaction was carried out at room temperature (~25 °C) for 5 min. After incubation, the resulting solution was analyzed by recording its UV-vis absorption spectrum using a UV-vis spectrophotometer, and the corresponding color change was documented photographically. These reaction conditions were selected based on the previously reported optimized protocols for Pt-based peroxidase nanozymes [46,47].

### 2.4. Steady-State Kinetic Analyses

All steady-state kinetic measurements were conducted according to our recent reports, with some modifications [46,47]; where the reaction temperature was room temperature, the reaction vessel was a cuvette with an optical path length of 1.0 cm, the reaction buffer was a citrate–phosphate buffer (pH 4.0), the catalyst was Au@PtNPs with a final concentration of 3.78 × 10^−14^ M, and the substrates were the reaction buffers containing 7.0 M H_2_O_2_ and TMB at varying concentrations in the range of 0.02–0.8 mM (refer to the Supporting Information for the experimental details). Finally, the apparent steady-state kinetic parameters including the Michaelis constant (*K*_m_), maximal reaction velocity (*V*_max_), and *K*_cat_ were obtained.

### 2.5. Preparation of SA-Conjugated Au@Pt_4L_NPs (Denoted as “SA-Au@Pt_4L_NP Conjugates”)

The preparation of SA-Au@Pt_4L_NP conjugates was performed following a previously reported protocol with slight adjustments [48]. Initially, 8 mL of the synthesized Au@Pt_4L_NPs suspension (7.55 × 10^−11^ M, in DI water) was treated with a 0.1 M Na_2_CO_3_ aqueous solution to achieve a pH of 9.0. Then, the Au@Pt_4L_NPs suspension was added with 12 μL of 2 mg mL^−1^ SA in PBS buffer (pH 7.4, 10 mM phosphate-buffered saline buffer). Following a 30 min incubation at room temperature, the Au@Pt_4L_NPs suspension was further added with 1 mL of 10% BSA in PBST [PBS buffer (pH 7.4) containing 0.05% Tween 20]. Following a 1 h incubation period, the final products (i.e., SA-Au@Pt_4L_NP conjugates) were obtained via centrifugation, washed with PBST, and finally resuspended in 0.8 mL of PBST containing 0.05% NaN_3_ and 1% BSA for future use (7.55 × 10^−10^ M).

### 2.6. Detection of IL-12 Using Au@Pt_4L_NP-Enhanced CELISA

Unless otherwise specified, all the experimental procedures were performed at room temperature. To prepare the CAb-coated 96-well microplates for IL-12 detection, the following steps were performed: (i) the microplates were incubated for 12 h at 4 °C with 50 μL of anti-IL-12 CAb (2500 ng mL^−1^) dissolved in coating buffer; (ii) following incubation, the microplates were washed five times with washing buffer, after which 350 μL of blocking buffer was added to each well and allowed to incubate for 2 h; and (iii) after removing the blocking buffer, the microplates were dried under ambient conditions, and finally stored at 4 °C with desiccant until needed. In a standard detection procedure, IL-12 standards of varying concentrations were prepared in dilution buffer, and 100 µL of each standard and 50 µL of biotin-anti-IL-12 DAb (200 ng mL^−1^, in dilution buffer) were added to the individual wells. The microplates were then shaken for 2 h. After washing five times with washing buffer, 100 µL of SA-Au@Pt_4L_NP conjugates (7.55 × 10^−11^ M, in dilution buffer) was introduced, with shaking for 30 min. Subsequently, the microplates were washed again and treated with 100 µL of a substrate solution containing 7.0 M H_2_O_2_ and 0.8 mM TMB in citrate–phosphate buffer (pH 4.0). The reactions were incubated for 20 min with shaking. To terminate the reactions, the microplates were added with 50 µL of stop solution. Finally, the absorbance at 450 nm for each well was recorded using a microplate reader.

For comparison, the HRP-based CELISA was conducted following the same procedure to the above Au@Pt_4L_NP-enhanced CELISA, with two key modifications: (i) the SA-Au@Pt_4L_NP conjugates were substituted with SA-HRP conjugates, and (ii) the substrate solution was adjusted to contain 2.0 mM H_2_O_2_ and 0.8 mM TMB in the buffer.

## 3. Results and Discussion

### 3.1. Synthesis and Characterization of Au@Pt_4L_NPs

**Figure 1:**
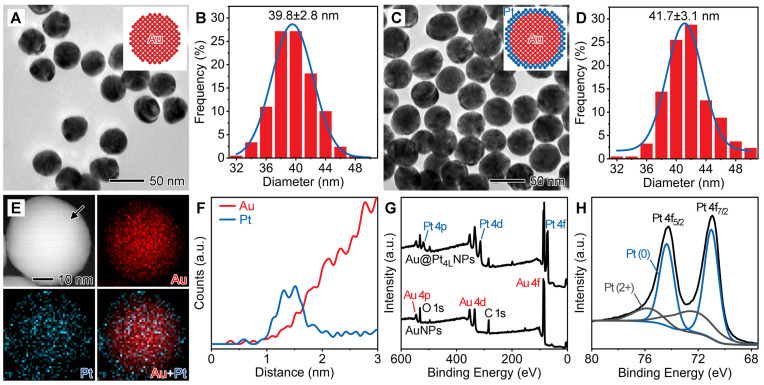
Characterization of Au@Pt_4L_NPs that were obtained from a standard synthesis. (**A**) Typical TEM images of ~40 nm AuNPs. Inset in (**A**) shows a corresponding 2D atomic model. (**B**) Distribution of diameter of the ~40 nm AuNPs based on counting more than 200 particles. (**C**) Typical TEM images of Au@Pt_4L_NPs. Inset in (**C**) shows a corresponding 2D atomic model. (**D**) Distribution of diameter of the Au@Pt_4L_NPs based on counting more than 200 particles. (**E**,**F**) EDX mapping image (**E**) and line-scan profiles (**F**) of an individual Au@Pt_4L_NPs (red = Au, blue = Pt). The EDX line-scan profiles were taken from a single nanoparticle shown in (**E**) along a direction as indicated by the arrow. (**G**) XPS survey spectra of AuNPs and Au@Pt4LNPs. (**H**) High-resolution XPS spectra of the Pt 4f region in Au@Pt4LNPs.

This study was initiated with the synthesis of Au@Pt_4L_NPs via a simple seed-mediated growth approach. Citrate-stabilized ~40 nm AuNPs were chosen as the seeds due to their widespread application in bio-assays and ease of synthesis in high quality via established protocols, enabling the efficient production of high-quality Au@Pt_4L_NPs. As observed in a transmission electron microscopy (TEM) image of the synthesized ~40 nm AuNPs shown in Figure 1A, the AuNPs exhibited uniform spherical morphology with smooth surfaces, and their diameter averaged 39.8 nm based on measurements of 200 individual particles (Figure 1B).

Figure 1C displays a representative TEM image of Au@Pt_4L_NPs synthesized using the standard procedure. The synthesized Au@Pt_4L_NPs displayed the same spherical morphology and smooth surface as the AuNP seeds, indicating a conformal coating of Pt on AuNPs. Their average diameter was measured to be 41.7 nm (Figure 1D), showing a 1.9 nm increment compared to the initial AuNP seeds. This corresponds to an estimated Pt shell thickness of ~0.95 nm. Differentiating Pt from Au using TEM alone is challenging due to their comparable lattice parameters and atomic masses [36]. Thus, energy-dispersive X-ray (EDX) analysis was employed for elemental mapping and line-scanning of Au and Pt elements in this Au@Pt_4L_NPs sample. As shown in the EDX mapping image (Figure 1E) of a single Au@Pt_4L_NP, Au (red) was localized within the particle core, while Pt (green) formed a continuous shell, confirming an Au@Pt core@shell nanostructure. Additionally, the EDX line scan of a single Au@Pt_4L_NP (Figure 1F) revealed Pt enrichment in the shell region, further supporting the conformal coating of Pt on AuNPs.

To further confirm the surface composition and chemical state of Pt in the Au@Pt_4L_NPs, an X-ray photoelectron spectroscopy (XPS) analysis was performed. As shown in Figure 1G, the XPS survey spectra revealed the presence of both Au and Pt in Au@Pt_4L_NPs, whereas only Au was detected in the original AuNP seeds, confirming the successful deposition of Pt onto AuNPs. The high-resolution Pt 4f spectra (Figure 1H) showed the coexistence of metallic Pt^0^ and oxidized Pt^2+^ species, with Pt^0^ being predominant, indicating that the deposited Pt primarily exists in its elemental state, which is essential for the catalytic activity. In addition, the crystallographic characterization of Au@Pt_4L_NPs was carried out using X-ray diffraction (XRD). As shown in Figure S1A, all diffraction peaks of Au@Pt_4L_NPs could be indexed to the face-centered cubic (*fcc*) structure of metallic Au (JCPDS no. 04-0784), with no distinct peaks corresponding to crystalline Pt, indicating that the Pt was deposited as thin shells on the Au cores without forming separate Pt nanoparticles [49]. Compared to the original AuNP seeds, the diffraction peaks of Au@Pt_4L_NPs exhibited a slight shift toward higher angles (Figure S1B), which is due to slight lattice contraction or interfacial strain at the Au-Pt boundary caused by lattice mismatch [50]. These observations further support the formation of conformal, ultrathin Pt shells on the surface of AuNPs.

To precisely evaluate the average number (*n*) of Pt atomic layers in the Pt shell for this Au@Pt_4L_NPs sample, inductively coupled plasma-optical emission spectrometry (ICP-OES) analysis was employed to determine the concentrations of Pt and Au elements for this Au@Pt_4L_NPs sample. The analysis revealed a Pt-to-Au molar ratio of 0.163:1. Using this ICP-OES analysis result, along with the measured size of the AuNP seeds (41.7 nm) and the known densities of Au (19.32 g/cm^3^) and Pt (21.45 g/cm^3^), the thickness of the Pt shell was calculated to be 0.92 nm. This value closely matches the 0.95 nm thickness obtained from TEM-based diameter measurements. Because ICP-OES analyzes a broader population of particles than TEM, it provides a more representative measurement of the Pt shell thickness and thus would be used in the following discussion. Because both Au and Pt possess a structure, where the Pt{111} planes have an interplanar spacing of 0.23 nm [36,51], the Pt shell of this Au@Pt_4L_NPs sample corresponds to approximately four Pt atomic layers. These results together demonstrate the successful synthesis of Au@PtNPs with ultrathin Pt shells of only four atomic layers.

### 3.2. Peroxidase-like Catalytic Properties of Au@Pt_4L_NPs

**Figure 2:**
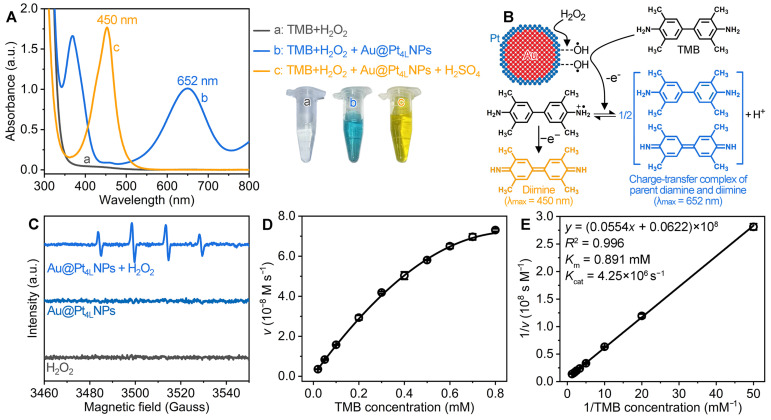
Peroxidase-like catalytic properties of Au@Pt_4L_NPs. (**A**) UV-vis spectra taken from H_2_O_2_-TMB solution catalyzed by Au@Pt_4L_NPs after a reaction time of 5 min: (a) H_2_O_2_-TMB substrate solution, (b) H_2_O_2_-TMB + Au@Pt_4L_NPs solution, and (c) H_2_O_2_-TMB + Au@Pt_4L_NPs + H_2_SO_4_ solution. The corresponding photographs of the three solutions are shown to the right of the spectra. (**B**) Schematic illustration showing the Au@Pt_4L_NP-catalyzed oxidation of TMB by H_2_O_2_ to generate oxTMB as the products. (**C**) ESR spectra of Au@Pt_4L_NPs + H_2_O_2_, Au@Pt_4L_NPs alone, and H_2_O_2_ alone, recorded using DMPO as a spin-trapping agent. (**D**,**E**) Steady-state kinetic evaluation of the peroxidase-like catalytic efficiency of Au@Pt_4L_NPs: (**D**) plot of *v* versus TMB concentration and (**E**) double-reciprocal plot derived from (**D**).

We then examined the peroxidase-like catalytic activity of Au@Pt_4L_NPs through their ability to catalyze the oxidation of TMB in the presence of H_2_O_2_. As depicted in Figure 2A, upon adding Au@Pt_4L_NPs to the substrate solution containing TMB and H_2_O_2_, the solution transitioned from colorless to blue, with an emergence of a characteristic absorption peak at 652 nm, which corresponds to the formation of a charge-transfer complex between the parent diamine (reduced TMB) and its oxidized diimine form (see Figure 2B for the molecular structures) [52]. Further, upon quenching the reaction with H_2_SO_4_, the solution turned from blue to yellow, accompanied by a shift in the absorption peak from 652 nm to 450 nm, which corresponds to the fully oxidized diimine product (see Figure 2B for the molecular structure) [52]. The findings reveal that Au@Pt_4L_NPs could effectively catalyze the oxidation of TMB by H_2_O_2_, leading to the production of oxidized TMB (oxTMB). This observation confirms the inherent peroxidase-like catalytic activity of Au@Pt_4L_NPs.

Previous work has shown that the underlying catalytic mechanism for the peroxidase-like catalytic activity of Pt-based peroxidase nanozymes involves the decomposition of H_2_O_2_ on the Pt surface to generate reactive oxygen species (ROS), particularly hydroxyl radicals (•OH), which are responsible for the oxidation of TMB to oxTMB [53,54,55]. To verify whether Au@Pt_4L_NPs follow a similar mechanism, electron spin resonance (ESR) spectroscopy was performed using 5,5-Dimethyl-1-pyrroline N-oxide (DMPO) as a spin-trapping agent. As shown in Figure 2C, a characteristic quartet ESR signal corresponding to DMPO–•OH adducts was observed in the Au@Pt_4L_NPs + H_2_O_2_ system, confirming the generation of •OH radicals. In contrast, no such signal was detected in the presence of Au@Pt_4L_NPs or H_2_O_2_ alone, indicating that •OH formation only occurs when both components are present. These results demonstrate that the peroxidase-like catalytic activity of Au@Pt_4L_NPs arises from their ability to decompose H_2_O_2_ and generate •OH, which subsequently oxidizes TMB (Figure 2B).

Further, the catalytic efficiency of Au@Pt_4L_NPs was quantified by measuring their catalytic constant (*K*_cat_), which represents the maximum number of oxTMB molecules produced per second per catalyst, using an apparent steady-state kinetic assay. A series of *ν* for the Au@Pt_4L_NPs-catalyzed reaction were initially measured for various TMB concentrations while fixing the H_2_O_2_ concentration at 7.0 M. Then, plotting ν against TMB concentration yielded a classic Michaelis–Menten curve (Figure 2B). These data were subsequently transformed into a double-reciprocal plot to calculate key kinetic parameters (Figure 2C). A linear relationship was observed between the reciprocal of ν and the reciprocal of TMB concentration, which is consistent with the Lineweaver–Burk equation, as follows:

$$\frac{1}{v}=\frac{K_{m}}{V_{max}}\times \frac{1}{\left[S\right]}+\frac{1}{V_{max}}$$

The *K*_m_ and *V*_max_ of Au@Pt_4L_NPs were obtained through the linear fitting regression analysis of this double-reciprocal plot. The *K*_cat_ was calculated via the following equation:

$$K_{cat}=\frac{V_{max}}{\left[E\right]}$$

As listed in Table 1, the *K*_cat_ value of Au@Pt_4L_NPs was determined to be 4.25 × 10^6^ s^−1^, which exceeded that of HRP by over 1063-fold, indicating the significantly enhanced catalytic efficiency of Au@Pt_4L_NPs compared to HRP. More significantly, Au@Pt_4L_NPs outperformed most previously reported peroxidase nanozymes in terms of *K*_cat_, further demonstrating their outstanding catalytic efficiency (Table 1). To assess Pt utilization efficiency, we calculated the mass-specific catalytic efficiency (*K*_cat-mass-specific_, s^−1^ mg^−1^_Pt_) of Au@Pt_4L_NPs by normalizing *K*_cat_ to the Pt content per nanocatalyst. The *K*_cat-mass-specific_ values for other Pt-based peroxidase nanozymes were similarly derived to enable a consistent and fair comparison. As shown in Table 1, although certain nanostructures such as concave Pt cubes (*K*_cat_ = 6.0 × 10^6^ s^−1^) showed higher *K*_cat_ values, their *K*_cat-mass-specific_ values were lower. For example, the 44 nm concave Pt cubes presented a *K*_cat-mass-specific_ value of only 3.28 × 10^18^ s^−1^ mg^−1^_Pt_, which is over 12-fold lower than that of Au@Pt_4L_NPs (*K*_cat-mass-specific_ = 4.13 × 10^19^ s^−1^ mg^−1^_Pt_), highlighting the superior Pt atom utilization efficiency of our Au@Pt_4L_NPs. Compared to small Pt nanoparticles such as 5–7 nm Pt particles (*K*_cat_ = 2.3 × 10^4^ s^−1^ and *K*_cat-mass-specific_ = 1.64 × 10^19^ s^−1^ mg^−1^_Pt_), our Au@Pt_4L_NPs achieved a higher *K*_cat_ value (4.25 × 10^6^ s^−1^) while maintaining a comparable *K*_cat-mass-specific_ value (4.13 × 10^19^ s^−1^ mg^−1^_Pt_), demonstrating an optimal balance between catalytic efficiency and Pt usage. This performance reflects the rational structural design of Au@Pt_4L_NPs, which integrates a large accessible surface area with highly dispersed Pt atoms, thereby maximizing active site exposure and enhancing mass-specific catalytic activity. In addition to high activity, Au@Pt_4L_NPs also exhibited good chemical and thermal stability, maintaining catalytic function after 2 h exposure to the pH range of 4–12 and the temperature range of 5–90 °C, as shown in Figure S2. The reduced stability observed at pH values below 4 or above 12 is due to the protonation or excessive deprotonation of citrate ions, which weakens the electrostatic repulsion and leads to nanoparticle aggregation [56]. Collectively, these findings establish Au@Pt_4L_NPs as a highly efficient and robust peroxidase nanozyme system with exceptional catalytic performance.

### 3.3. Influence of Pt Content on the Activity of Au@PtNPs

**Figure 3:**
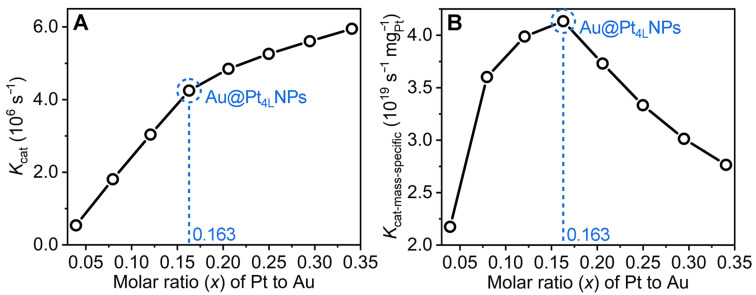
Influence of Pt content on the peroxidase-like catalytic efficiency of Au@PtNPs. (**A**,**B**) Two plots comparing the *K*_cat_ (**A**) and *K*_cat-mass-specific_ (**B**) values of Au@PtNPs with different Pt-to-Au molar ratios (*x*, denoted as Au@Pt*_x_*NPs, *x* = 0.039–0.341). Au@Pt_0.163_NPs correspond to the Au@Pt_4L_NPs shown in Figure 1C.

To investigate how Pt content impacts the peroxidase-like catalytic efficiency of Au@PtNPs, a series of Au@PtNPs with varying Pt-to-Au molar ratios (*x*, denoted as Au@Pt*_x_*NPs, where *x* ranges from 0.039 to 0.341) were synthesized by adjusting the quantity of K_2_PtCl_6_ added into the standard synthesis procedure. The peroxidase-like catalytic efficiencies of these Au@PtNPs were evaluated using the apparent steady-state kinetic assays. Figure 3A illustrates the variation in *K*_cat_ values of these Au@Pt*_x_*NPs across the *x* range of 0.039–0.341. A significant increase in *K*_cat_ value was observed as *x* rose from 0.039 to 0.163, reaching 4.25 × 10^6^ s^−1^. As *x* further increased to 0.341, the *K*_cat_ value continued to rise steadily, reaching 5.95 × 10^6^ s^−1^. These findings suggest a strong positive correlation between Pt content and catalytic efficiency within the studied range. Additionally, the mass-specific catalytic efficiencies (*K*_cat-mass-specific_) were also analyzed for these Au@Pt*_x_*NPs. As shown in Figure 3B, the *K*_cat-mass-specific_ value initially increased from 2.17 × 10^19^ s^−1^ mg^−1^_Pt_ to 4.13 × 10^19^ s^−1^ mg^−1^_Pt_ as *x* increased in the range of 0.039 to 0.163. Subsequently, it decreased from 4.13 × 10^19^ s^−1^ mg^−1^_Pt_ to 2.77 × 10^19^ s^−1^ mg^−1^_Pt_ with a further increase in *x* within the range of 0.163 to 0.341, exhibiting a volcano-shaped trend. This trend indicates that Au@Pt_0.163_NPs (corresponding to the Au@Pt_4L_NPs depicted in Figure 1C) achieve the most effective balance between the catalytic efficiency and the Pt utilization, positioning them as the optimal configuration for further experimentation. This efficiency is particularly valuable given the scarcity and high cost of Pt. The exceptional catalytic performance of Au@Pt_4L_NPs can be attributed to the distinctive surface electronic structures of the Pt atomic layers on the Au surface, which arise from the synergistic interaction between the Pt and Au components.

### 3.4. Preparation and Verification of SA-Au@Pt_4L_NP Conjugates

**Figure 4:**
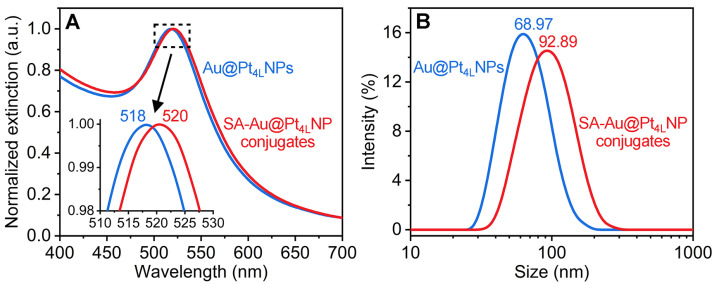
Characterization of SA-Au@Pt_4L_NP conjugates. (**A**) Normalized UV-vis extinction spectra of Au@Pt_4L_NPs (blue) and SA-Au@Pt_4L_NP conjugates (red) dispersed in DI water. (**B**) DLS size distributions of Au@Pt_4L_NPs (blue) and SA-Au@Pt_4L_NP conjugates (red) dispersed in DI water.

To enable the utilization of Au@Pt_4L_NPs in CELISA of IL-12, these nanoparticles were functionalized with SA, yielding SA-Au@Pt_4L_NP conjugates that served as signal probes. To verify the successful formation of SA-Au@Pt_4L_NP conjugates, UV-vis spectroscopy was employed to examine the Au@Pt_4L_NPs before and after SA modification. As shown in Figure 4A, the major extinction peak of Au@Pt_4L_NPs exhibited a red shift from 518 nm to 520 nm following SA modification, implying the binding of SA molecules to the surface of Au@Pt_4L_NPs [65,66]. Additionally, dynamic light scattering (DLS) measurements were conducted to assess the hydrodynamic size distribution of Au@Pt_4L_NPs before and after SA modification. As shown in Figure 4B, the average hydrodynamic diameter of Au@Pt_4L_NPs increased significantly from 68.97 nm to 92.89 nm after SA modification, further suggesting the successful attachment of SA biomolecules to the surface of Au@Pt_4L_NPs [67,68]. The interaction mechanisms underlying the conjugation of SA protein to Au@Pt_4L_NP surface primarily involve three types of binding: electrostatic attraction between the negatively charged surface of Au@Pt_4L_NPs and the positively charged domains on SA protein, hydrophobic interactions where the protein’s hydrophobic regions associate with the nanoparticle surface, and the potential covalent bonding of Pt atoms with thiol groups present in SA protein [48]. Together, these observations confirm the successful preparation of SA-Au@Pt_4L_NP conjugates.

### 3.5. Analytical Performance of Au@Pt_4L_NP-Enhanced CELISA

To implement Au@Pt_4L_NPs in a CELISA system for IL-12 detection, these nanoparticles were employed as alternative signal tags, replacing traditional HRP labels. This innovation aimed to establish a highly sensitive and advanced CELISA platform (referred to as Au@Pt_4L_NP-enhanced CELISA, Scheme 1). The evaluation process focused on assessing its analytical performance, including sensitivity and reproducibility.

**Figure 5:**
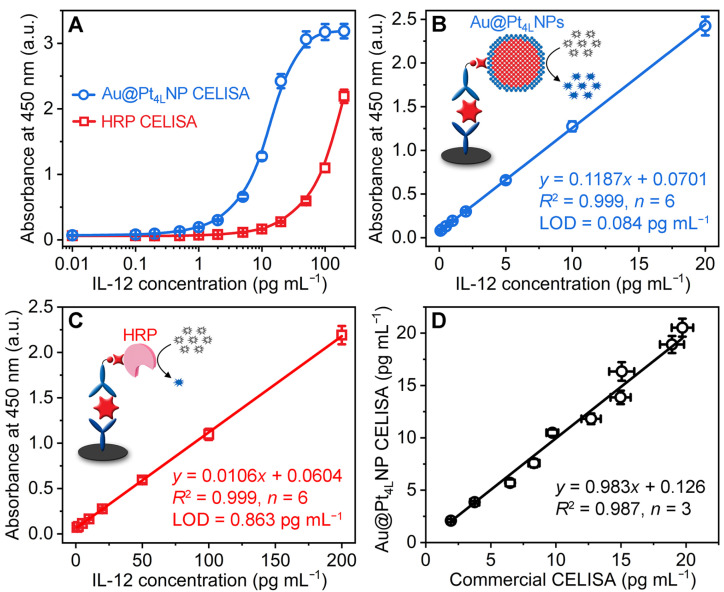
Detection of IL-12 using the Au@Pt_4L_NP-enhanced CELISAs. (**A**–**C**) Detection of IL-12 standards using the Au@Pt_4L_NP-enhanced (blue) and HRP-based (red) CELISAs: (**A**) Calibration curves of the two CELISAs that were generated by plotting the absorbance at 450 nm against IL-12 concentration; (**B**,**C**) corresponding linear ranges of the calibration curves: Au@Pt_4L_NP-enhanced CELISA (**B**) and HRP-based CELISA (**C**). Note: error bars indicate intra-batch variation. (**D**) Correlation analysis of IL-12 levels in 10 serum samples measured by the Au@Pt_4L_NP-enhanced CELISA and a commercial HRP-based CELISA kit.

To determine sensitivity, a standard calibration curve was constructed using a series of diluted IL-12 standards with varying concentrations. The absorbance values at 450 nm were plotted against the corresponding IL-12 concentrations to derive the calibration curve and its linear range. From this, the slope of the regression curve was calculated to assess sensitivity. The LOD was calculated as 3SD divided by this slope, with SD representing the standard deviation of the blank measurements obtained from intra-batch variation. For comparison, the sensitivity and LOD of the conventional HRP-based CELISA system were also evaluated under the same conditions. As illustrated in Figure 5A, the Au@Pt_4L_NP-enhanced CELISA exhibited a proportional increase in absorbance at 450 nm across a concentration range of 0.1–100 pg mL^−1^, covering three orders of magnitude. Compared to the HRP-based CELISA, which displayed a narrower range of 1–200 pg mL^−1^, the Au@Pt_4L_NP-enhanced CELISA platform provided a wider detection window. Furthermore, the Au@Pt_4L_NP-enhanced CELISA produced significantly higher absorbance signals than the HRP system, confirming its enhanced sensitivity. To further quantify the platform’s sensitivity, the linear range of the calibration curve was examined. As shown in Figure 5B, a high-quality linear relationship between the absorbance and IL-12 concentration was observed from 0.1 to 20 pg mL^−1^, with an excellent correlation coefficient (*R*^2^ = 0.999, *n* = 6). The regression slope and LOD were calculated as 0.1187 a.u. mL pg^−1^ and 0.084 pg mL^−1^ (1.1 fM), respectively, representing a ~10-fold improvement over the HRP-based CELISA system (0.0106 a.u. mL pg^−1^ and 0.863 pg mL^−1^, Figure 5C). Remarkably, the LOD achieved was at the fM level, surpassing the LODs of commercially available IL-12 CELISA kits (Table S1). This dramatic improvement can be attributed to the exceptional catalytic efficiency of Au@Pt_4L_NPs compared to HRP.

The reproducibility of the Au@Pt_4L_NP-enhanced CELISA was assessed by analyzing the variabilities of intra- and inter-assays. IL-12 standards at low (0.5 pg mL^−1^), medium (5 pg mL^−1^), and high (50 pg mL^−1^) concentrations were measured in triplicate, using the same and different batches of Au@Pt_4L_NPs. The coefficient of variation (CV, *n* = 6) was calculated to quantify consistency. The results, summarized in Table S3, showed that the intra-assay CV values were consistently below 5.08%, while inter-assay CV values remained under 7.36%, demonstrating the platform’s high reproducibility. This consistency supports the potential for large-scale production and its suitability for clinical applications.

### 3.6. Application in Analysis of Serum Samples

To assess the suitability of the Au@Pt_4L_NP-enhanced CELISA for clinical applications, the CELISA was employed to detect IL-12 in seven serum samples spiked with known concentrations of IL-12. The samples were prepared by spiking fetal bovine serum (FBS), which inherently lacks human IL-12, with IL-12 at final concentrations of 0.2, 0.5, 1, 2, 5, 10, and 20 pg mL^−1^. This choice of matrix ensured that potential interference from endogenous human IL-12 present in human serum was eliminated. The IL-12 concentrations in the spiked samples were calculated using the linear curves depicted by the blue curves in Figure 5B. To validate the method, recovery analysis was performed by comparing the measured IL-12 concentrations to the spiked concentrations. As shown in Table 2, the recoveries ranged from 92.53% to 105.55% with CV values below 8.90% (*n* = 3) for all six samples. These findings demonstrate that the Au@Pt_4L_NP-enhanced CELISA exhibits exceptional accuracy and consistency, even in the presence of complex serum matrices, confirming the assay’s strong potential for reliable quantification of trace levels of IL-12 in real biological samples. To further assess the reliability of the developed immunoassay under blinded conditions, a double-blind test was conducted. In this test, one student prepared serum samples by spiking FBS with IL-12 at predefined but undisclosed concentrations, while another student, blinded to the actual values, analyzed the samples using the Au@Pt_4L_NP-enhanced CELISA. The detected IL-12 concentrations were then compared with those obtained using a commercial HRP-based IL-12 CELISA kit (from ThermoFisher Scientific, Inc.) as the reference method. As shown in Figure 5D, the linear regression analysis revealed a strong positive correlation (*R*^2^ = 0.987) between the two methods, with the fitted line exhibiting a slope close to unity (0.983) and a small intercept (0.126). These findings further confirm the robustness, accuracy, and practical applicability of the Au@Pt_4L_NP-enhanced CELISA for the reliable detection of IL-12 in complex biological matrices.

## 4. Conclusions

In summary, we have developed an innovative CELISA platform with high sensitivity for detecting IL-12 at femtomolar levels through the strategic design of Au@Pt_4L_NPs as signal labels. These Au@Pt_4L_NPs exhibit exceptional peroxidase-like catalytic activity while minimizing Pt usage, ensuring both high efficiency and cost-effectiveness. Specifically, they achieve a *K*_cat_ reaching up to 4.25 × 10^6^ s^−1^, ~1063 times higher than that of HRP. Moreover, among a series of Au@PtNPs with Pt shells of different contents, the Au@Pt_4L_NPs with a Pt-to-Au molar ratio of 0.163 display the highest mass-specific catalytic activity. In addition, they can be produced with high quality, making them reliable for large-scale applications. Owing to these features, the Au@Pt_4L_NP-enhanced CELISA offers several important advantages for IL-12 detection: (i) it achieves femtomolar sensitivity with a LOD of 0.084 pg mL^−1^ (1.1 fM), enabling the precise quantification of IL-12 at extremely low concentrations; (ii) it demonstrates excellent reproducibility, ensuring consistent performance; and (iii) it is scalable, supporting broader clinical and research applications. To our knowledge, this study represents the first successful demonstration of a CELISA capable of femtomolar sensitivity for IL-12 detection using a single-step signal amplification strategy. Compared to the conventional HRP-based CELISA, our platform achieves a LOD that is ~10-fold lower, allowing for the accurate and reliable monitoring of trace levels of IL-12 in complex biological matrices such as serum. This work establishes the Au@Pt_4L_NP-enhanced CELISA as a highly promising tool for in vitro diagnostics, offering new possibilities for the quantitative detection of low-abundance biomarkers in clinical and biomedical research. By addressing the sensitivity limitations of traditional colorimetric assays, our findings open up new opportunities for applying nanomaterials in biosensing, paving the way for the development of next-generation diagnostic technologies with unparalleled performance.

## Figures and Tables

**Table 1:** Comparison of the kinetic parameters of various catalysts toward the catalysis of the H_2_O_2_-TMB reaction.

Catalyst	Size (nm)	[*E*] (M)	Substance	*K*_m_ (M)	*V*_max_ (M s^−1^)	*K*_cat_ (s^−1^)	*K*_cat-mass-specfic_ (s^−1^ mg^−1^_Pt_)	Refs.
HRP	N/A	2.5 × 10^−11^	TMB	4.3 × 10^−4^	1.0 × 10^−7^	4.0 × 10^3^	N/A	[23]
Fe_3_O_4_ particles	300	1.1 × 10^−12^	TMB	9.8 × 10^−5^	3.4 × 10^−8^	3.0 × 10^4^	N/A	[23]
Co_3_O_4_ cubes	20	3.4 × 10^−10^	TMB	3.7 × 10^−5^	6.3 × 10^−8^	1.8 × 10^2^	N/A	[57]
MnO_2_ particles	4.5	3.0 × 10^−8^	OPD	3.1 × 10^−4^	8.2 × 10^−8^	2.7 × 10^0^	N/A	[58]
V_2_O_5_ wires	100 × 500	1.1 × 10^−4^	ABTS	4.0 × 10^−7^	2.8 × 10^−1^	2.5 × 10^3^	N/A	[59]
Au particles	40	6.7 × 10^−12^	TMB	N/A	4.8 × 10^−8^	7.2 × 10^3^	N/A	[36]
Ru frames	10	1.1 × 10^−12^	TMB	6.0 × 10^−5^	1.3 × 10^−7^	1.3 × 10^4^	N/A	[60]
Au@Pt rods	30 × 70	1.3 × 10^−11^	TMB	2.7 × 10^−5^	1.8 × 10^−7^	1.4 × 10^4^	N/A	[53]
Pt particles	5–7	8.1 × 10^−11^	TMB	1.2 × 10^−4^	1.3 × 10^−6^	2.3 × 10^4^	1.64 × 10^19^	[54]
Pd cubes	18	1.4 × 10^−12^	TMB	5.4 × 10^−5^	9.7 × 10^−8^	6.9 × 10^4^	N/A	[61]
Pd-Ru cubes	20	N/A	TMB	N/A	N/A	4.8 × 10^5^	N/A	[62]
Pt cubes	7.4	4.1 × 10^−13^	TMB	7.3 × 10^−4^	3.3 × 10^−7^	8.2 × 10^5^	9.63 × 10^19^	[63]
Pd-Ir cubes	19.2	3.4 × 10^−14^	TMB	1.3 × 10^−4^	6.5 × 10^−8^	1.9 × 10^6^	N/A	[61]
Pd@Pt cubes	20	2.6 × 10^−13^	TMB	3.4 × 10^−4^	6.0 × 10^−7^	2.3 × 10^6^	4.40 × 10^19^	[64]
Concave Pt cubes	44	2.5 × 10^−14^	TMB	N/A	1.5 × 10^−7^	6.0 × 10^6^	3.28 × 10^18^	[47]
Pd@Pt dendrites	27	2.44 × 10^−14^	TMB	6.63 × 10^−4^	2.21 × 10^−7^	9.1 × 10^6^	8.20 × 10^19^	[46]
Au@Pt_4L_NPs	42	3.78 × 10^−14^	TMB	8.91 × 10^−4^	1.61 × 10^−7^	4.25 × 10^6^	4.13 × 10^19^	This work

**Table 2:** Analytical recoveries of Au@Pt_4L_NP-enhanced CELISA in the analysis of IL-12-spiked serum samples.

Sample No.	Spiked (pg mL^−1^)	Found (Mean ± SD) (pg mL^−1^)	CV (%, *n* = 3)	Recovery (%)
1	0.2	0.185 ± 0.015	8.30	92.53
2	0.5	0.479 ± 0.025	5.25	95.87
3	1	1.043 ± 0.093	8.90	104.30
4	2	2.055 ± 0.176	8.57	102.75
5	5	5.118 ± 0.280	5.47	102.36
6	10	10.555 ± 0.534	5.06	105.55
7	20	19.666 ± 1.038	5.28	98.33

## Data Availability

The data that support the findings of this study are available from the corresponding authors upon reasonable request.

## Supplementary Materials

The following supporting information can be downloaded at https://www.mdpi.com/article/10.3390/bios15040239/s1: Table S1: Comparison of the limits of detection (LODs) for IL-12 detection using different commercial ELISA kits; Table S2: Dosages of reagents used for synthesizing various Au@PtNPs with different Pt-to-Au molar ratios (*x*, denoted as Au@Pt*x*NPs, where *x* ranges from 0.039 to 0.339); Table S3: Intra- and inter-batch coefficients of variation (CVs, *n* = 6) of the Au@Pt_4L_NP-enhanced CELISA in detecting 0.5, 5, and 50 pg mL^−1^ IL-12 standards. Figure S1: (A) XRD patterns of the as-synthesized 40 nm AuNPs (red) and Au@Pt_4L_NPs (blue), and (B) Magnified view of the corresponding (111), (200), (220), (311), and (222) diffraction peaks. Figure S2: Stability evaluation for the peroxidase-like catalytic activity of Au@Pt_4L_NPs. Relative catalytic activities of Au@Pt_4L_NPs after incubation with acid or base (pH 1–14) for 2 h (A) and treatment with heat (5–90 °C) for 2 h (B), in which the activity at pH 4.0 and 22 was set as 100%. References [44,46,47] are cited in the Supplementary Materials.

## Abbreviations

The following abbreviations are used in this manuscript:

CELISAs	Colorimetric enzyme-linked immunosorbent assays
AuNPs	Au nanoparticles
Au@PtNPs	(Au core)@(Pt shell) nanoparticles
HRP	Horseradish peroxidase
LOD	Limit of detection
IL-12	Interleukin-12
*K*_cat_	Catalytic constant
CAbs	Capture antibodies
DAbs	Detection antibodies
SA	Streptavidin
TMB	3,3′,5,5′-Tetramethylbenzidine
oxTMB	Oxidized TMB
H_2_O_2_	Hydrogen peroxide
TEM	Transmission electron microscopy
EDX	Energy-dispersive X-ray
ICP-OES	Inductively coupled plasma-optical emission spectrometry
*fcc*	Face-centered cubic
*K*_m_	Michaelis constant
*V*_max_	Maximal reaction velocity
ν	Initial reaction velocity
*K*_cat-mass-specific_	Mass-specific catalytic efficiency
SD	Standard deviation
CV	Coefficient of variation
FBS	Fetal bovine serum
DI	Deionized
PBS	Phosphate-buffered saline
PBST	PBS buffer (pH 7.4) containing 0.05% Tween 20
XPS	X-ray photoelectron spectroscopy
XRD	X-ray diffraction
ESR	Electron spin resonance
