# Supplementary material for: Atomic Pt-Layer-Coated Au Peroxidase Nanozymes with Enhanced Activity for Ultrasensitive Colorimetric Immunoassay of Interleukin-12
Source: Biosensors (Basel). 2025 Apr 9;15(4):239. doi: 10.3390/bios15040239 (PMC12025286; doi:10.3390/bios15040239)
Supplement: Supplementary file 1 [file biosensors-15-00239-s001.zip › biosensors-3515478-supplementary.pdf]

Supporting Information for

Atomic Pt-Layer-Coated Au Peroxidase Nanozymes with Enhanced Activity for Ultrasensitive Colorimetric Immunoassay of Interleukin-12

Han Zhang[1,2], Xiang Peng[2], Hao Song[2], Yongfeng Tan[2], Jianglian Xu[2], Qunfang Li[1,*] and Zhuangqiang Gao[2,*].

[1] School of Chemistry and Environmental Engineering, Hubei Minzu University, Enshi, 445000, People's Republic of China; 17852602023@163.com (H.Z.)
[2] Marshall Laboratory of Biomedical Engineering, Shenzhen Key Laboratory for Nano-Biosensing Technology, School of Biomedical Engineering, Shenzhen University Medical School, Shenzhen University, Shenzhen, Guangdong, 518060, People's Republic of China; 17852602023@163.com (H.Z.); zora1951@163.com (X.P.); 15249939659@163.com (H.S.); tanyongfeng0@gmail.com (Y.T.); jianglianxu0916@gmail.com (J.X.)
* Correspondence: slindar809@126.com (Q. L.) and zhuangqiang.gao@szu.edu.cn (Z. G.)

Additional Materials and Methods

S1. Chemicals and Materials

Gold(III) chloride trihydrate ($HAuCl_4·3H_2O$, ≥99.9%) and potassium hexachloroplatinate(IV) (K_2PtCl_6, 99.95%) were purchased from Shanghai Titan Scientific Co., Ltd (Shanghai,China). Sodium citrate dihydrate (≥99%) and L-ascorbic acid (AA, ≥99%) were purchased from Millipore Sigma (St. Louis, MO, USA). Sulfuric acid (H_2SO_4, 95~98%) was purchased from Xilong Scientific (Guangzhou,China). Sodium carbonate (Na_2CO_3, ≥99.5%), sodium bicarbonate ($NaHCO_3$, ≥99.7%), potassium phosphate monobasic (KH_2PO_4, ≥99%), sodium phosphate dibasic (Na_2HPO_4, ≥99%), sodium chloride (NaCl, ≥99.5%), potassium chloride (KCl, ≥99%), citric acid (≥99%), Tween 20, bovine serum albumin (BSA, ≥98%), and 3,3',5,5'-tetramethylbenzidine (TMB, ≥99%) were purchased from Aladdin (Shanghai,China). Sodium azide (NaN_3, ≥99.5%) and hydrogen peroxide solution (H_2O_2, 30 wt% in H_2O) were purchased from Sinopharm Chem. Re. Co., Ltd (Beijing, China). Human interleukin-12 (IL-12), mouse anti-IL-12 capture antibody, biotin-conjugated mouse anti-IL-12 detection antibody, streptavidin (SA), SA-conjugated horseradish peroxidase (HRP, SA-HRP), HRP-based IL-12 CELISA kit, and fetal bovine serum were obtained from ThermoFisher Scientific, Inc (Waltham, MA, USA). High-binding 96-well microtiter plates (No. 655061) were obtained from Greiner Bio-One (Kremsmünster, Austria). All aqueous solutions were prepared using deionized (DI) water with a resistivity of 18.2 MΩ·cm.

A 10 mM carbonate-bicarbonate buffer (pH 9.6) was prepared by dissolving 1.69 g Na_2CO_3 and 2.86 g $NaHCO_3$ in 1000 mL DI water. A 10 mM phosphate-buffered saline (PBS, pH 7.4) was prepared by dissolving 2.9 g $Na_2HPO_4·12H_2O$, 0.24 g KH_2PO_4, 0.2 g KCl, and 8.0 g NaCl in 1000 mL DI water. A citrate-phosphate buffer (pH 4.0) was prepared by dissolving 11.825 g Na_2HPO_4 and 2.365 g citric acid in 1.0 L of DI water. The washing buffer was prepared by adding 0.05% (v/v) Tween 20 in PBS (pH 7.4, PBST). The block-fix buffer was prepared by adding 1.0% (w/v) BSA and 15% (w/v) sucrose in PBST. The dilution buffer was prepared by adding 1.0% (w/v) BSA in PBST.

S2. Characterizations

The transmission electron microscope (TEM) images were taken using a HITACH HT7700 TEM (Hitachi High-Tech, Tokyo, Japan) operated at 100 kV. The energy

 https://doi.org/10.3390/xxxxx

dispersive X-ray (EDX) mapping and EDX line-scan profile were acquired using a JEOL F200 TEM (JEOL Ltd., Tokyo, Japan) operated at 200 kV. The amounts of Au and Pt elements in various nanostructures were determined using an inductively coupled plasma-optical emission spectrometry (ICP-OES, an Avio 220 Max ICP-OES Scott/Cross-Flow Configuration, PerkinElmer, Waltham, MA, USA). The X-ray photoelectron spectroscopy (XPS) measurements were performed on a PHI 5000 VersaProbe III XPS system (ULVAC-PHI, Kanagawa, Japan). The X-ray diffraction (XRD) patterns were recorded using a Bruker D8 VENTURE diffractometer (Bruker AXS GmbH, Karlsruhe, Germany). The photographs of samples in tubes were taken using the camera of an iPhone 14 (Apple Inc., Cupertino, CA, USA). The UV-vis absorption and extinction spectra and kinetic curves of apparent steady-state kinetic assays were recorded using a SHIMADZU UV-2600i UV-vis spectrophotometer (Shimadzu Corporation, Kyoto, Japan). The electron spin resonance (ESR) spectra were recorded using a Bruker EMXplus spectrometer (Bruker BioSpin GmbH, Rheinstetten, Germany). The dynamic light scattering (DLS) data were obtained using a Zetasizer Pro Particle Size and Zeta Potential Analysis System (Malvern Panalytical Ltd., Malvern, UK). The absorbance of samples in wells of microplates was measured using a BioTek EPOCH2 microplate reader (Agilent Technologies, Winooski, VT, USA). The pH values of different buffers were measured using an OHAUS STARTER3100 meter (OHAUS Corporation, Parsippany, NJ, USA).

S3. Preparation of ~15 nm AuNPs

~15 nm AuNPs were synthesized *via* a modified version of the classic Frens' method [44]. Briefly, 0.5 mL of an aqueous solution of $HAuCl_4$ (1%, w/v) and 47.5 mL of deionized (DI) water were added into a 100-mL round-bottom flask equipped with a reflux condenser. The solution was heated to boiling under continuous stirring in an oil bath. Once boiling, 2 mL of an aqueous solution of sodium citrate (1%, w/v) was quickly introduced into the boiling solution using a pipette. The mixed solution was maintained at boiling temperature with stirring for 30 min, during which the solution's color gradually transitioned to red, indicating the formation of ~15 nm AuNPs. The synthesized ~15 nm AuNPs suspension was then cooled to room temperature (~25°C) and stored in a dark environment for future use. The final product contained ~0.058 mg/mL of Au element.

S4. Steady-State Kinetic Analyses

All steady-state kinetic measurements were conducted according to our recent reports with some modifications [46,47]. The experiments were carried out at room temperature in cuvettes using a citrate-phosphate buffer (pH 4.0) as the reaction buffer. Au@PtNPs suspension was used at a final concentration of 3.78×10^{-14} M. Reactions were initiated by quickly adding nanoparticles into the reaction buffer containing 7.0 M H_2O_2 and TMB at varying concentrations in the range of 0.02-0.8 mM. The absorbance at 652 nm of each reaction solution was continuously recorded as a function of time for 3 min (5-second intervals) using a UV-vis spectrophotometer. To determine the initial reaction velocity, the initial slope of the "absorbance *vs* time" curve was extracted through the first derivative analysis using OriginPro 2021b software. The initial reaction velocity (ν) was calculated as:

$$v = \frac{\mathrm{Slope_{initial}}}{\varepsilon_{\mathrm{oxTMB-652\ nm}} \times l}$$

where $\varepsilon_{\mathrm{oxTMB\text{-}652\ nm}}$ is the molar extinction coefficient of oxidized TMB (oxTMB) at 652 nm, which is equal to 3.9×10^4 M^{-1} cm^{-1}, and l is the optical path length of the cuvette. A plot of ν versus TMB concentration was fitted to the Michaelis-Menten equation:

$$v = \frac{V_{max} \times [S]}{K_\mathrm{m} \times [S]}$$

where V_{max} is the maximal reaction velocity, $[S]$ is the TMB concentration, and K_m is the Michaelis constant. Finally, the apparent steady-state kinetic parameters were determined using:

$$\frac{1}{v} = \frac{K_m}{V_{max}} \times \frac{1}{[S]} + \frac{1}{V_{max}}$$

The catalytic constant (K_{cat}) was derived from the equation:

$$K_{cat} = \frac{V_{max}}{[E]}$$

where $[E]$ represents the catalyst concentration.

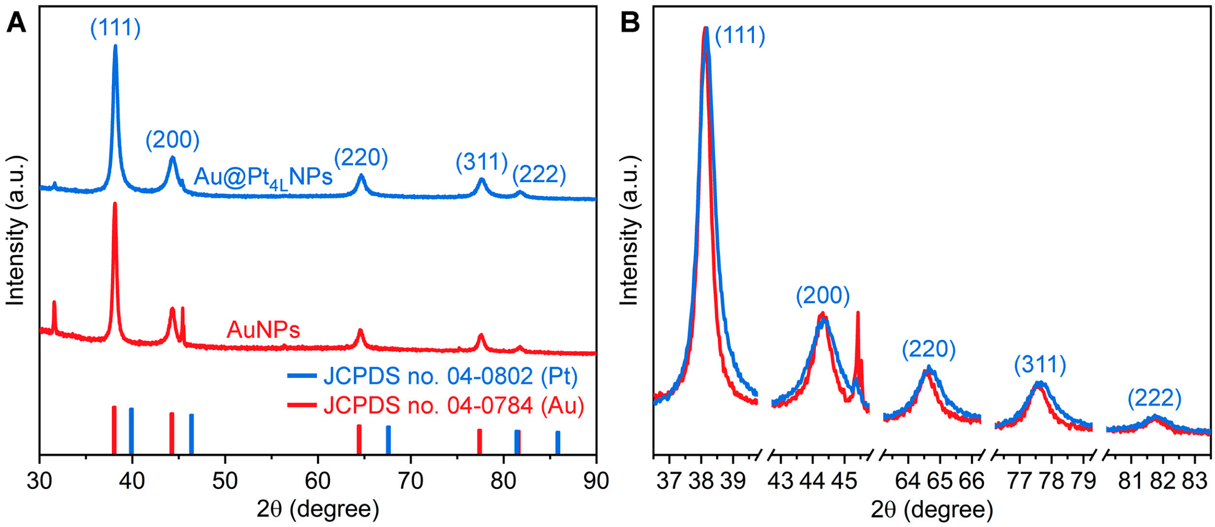

Figure S1. (A) XRD patterns of the as-synthesized 40 nm AuNPs (red) and Au@Pt_{4L}NPs (blue), and (B) Magnified view of the corresponding (111), (200), (220), (311), and (222) diffraction peaks.

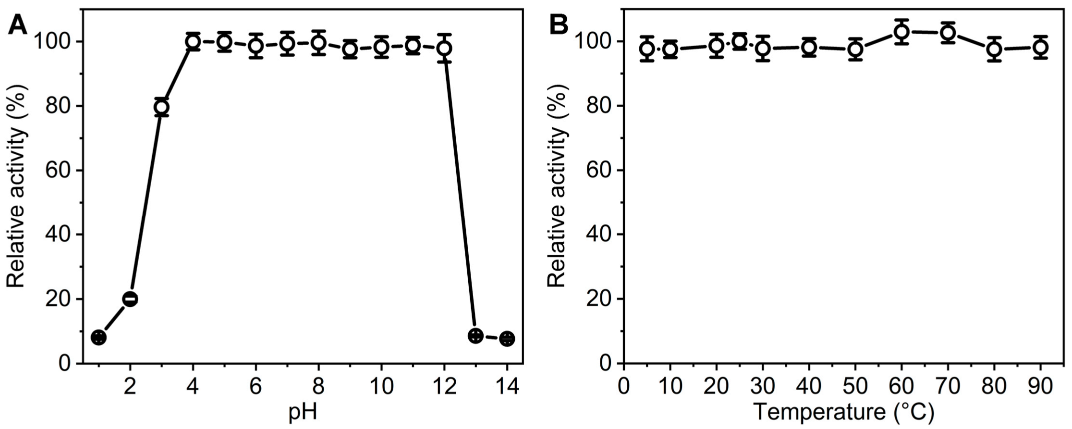

Figure S2. Stability evaluation for the peroxidase-like catalytic activity of Au@Pt_{4L}NPs. Relative catalytic activities of Au@Pt_{4L}NPs after incubation with acid or base (pH 1-14) for 2 h (A) and treatment with heat (5-90 °C) for 2 h (B), in which the activity at pH 4.0 and 22 was set as 100%.

Table S1. Comparison of the limits of detection (LODs) for IL-12 detection using different commercial ELISA kits.

Detection method	Manufacturer	Catalog number	Website	LOD (pg mL^{-1})	LOD (fM)
Commercial ELISA kits	Fisher Scientific	88-7126-88	https://www.ther-mofisher.com/elisa/product/Human-IL-12-p70-Uncoated-ELISA-Kit/88-7126-88	4.0	53.3
		BMS238	https://www.ther-mofisher.com/elisa/product/Human-IL-12-p70-ELISA-Kit/BMS238	2.1	28.0
	Abcam	ab46143	https://www.abcam.com/en-us/prod-ucts/elisa-kits/human-il-12-p70-elisa-kit-ab46143?srsltid=Afm-BOoo8vw7hpoSa2AN1PyJ_tIR_wDWiZ9tdt-LCItAeNKwzQvYcgnnM	2.2	29.3
		ab213791	https://www.abcam.com/en-us/prod-ucts/elisa-kits/human-il-12-p70-elisa-kit-ab213791?srsltid=AfmBOorzRM-CHmOeTYb3KXnUdR-wRwfNMSQivNfd-iyrmNMFp1zt_ucf0R3	2.0	26.7
		ab100552	https://www.abcam.com/en-us/prod-ucts/elisa-kits/human-il-12-p70-elisa-kit-ab100552?srsltid=Afm-BOop_VJQuEFQ-7X75sLPV8WxO6MZr0qMdn3y6TW41FFGWx4UVkZUZ	1.0	13.3
	Milllipore Sigma	RAB0252	https://www.sigmaal-drich.com/US/en/prod-uct/sigma/rab0252?srsltid=AfmBOor-geEY1nYZ_FPSVnWgdtxwqhram7uGjL727V8upkZsQbjiovGWD	1.0	13.3
	R&D Systems	D1200	https://www.rndsystems.com/prod-ucts/human-il-12-p70-quantikine-elisa-kit_d1200	2.95	39.3

Table S2. Dosages of reagents used for synthesizing various Au@PtNPs with different Pt-to-Au molar ratios (*x*, denoted as Au@Pt_xNPs, where *x* ranges from 0.039 to 0.339).

Pt-to-Au molar ratio (*x*)	Concentration of K_2PtCl_6 solution (mM)	Volume of K_2PtCl_6 solution (μL)	Volume of DI water (μL)
0.039	1	91	3909
0.080	1	185	3815
0.121	1	280	3720
0.163	1	378	3622
0.206	1	478	3522
0.250	1	580	3420
0.295	1	685	3315
0.341	1	791	3209

Table S3. Intra- and inter-batch coefficients of variation (CVs, $n = 6$) of the Au@Pt$_{4L}$NP-enhanced CELISA in detecting 0.5, 5, and 50 pg mL^{-1} IL-12 standards.

	IL-12 conc. (pg mL^{-1})	Absorbance at 450 nm (a.u.)						Mean (nm)	Standard deviation (SD, nm)	CV (%, n = 6)
		1	2	3	4	5	6			
Intra-batch assay	0.5	0.1307	0.1415	0.1225	0.1328	0.1269	0.1316	0.1310	0.0064	4.86
	5	0.7022	0.6841	0.6281	0.6798	0.6281	0.6314	0.6590	0.0335	5.08
	50	3.2349	3.0522	2.9281	3.0501	2.9422	3.1648	3.0621	0.1208	3.95
Inter-batch assay	0.5	0.1253	0.1467	0.1348	0.1516	0.1291	0.1401	0.1379	0.0102	7.36
	5	0.6065	0.6898	0.6305	0.7282	0.6199	0.6764	0.6586	0.0472	7.17
	50	2.8347	3.1351	3.0306	3.2957	2.9407	3.0509	3.0480	0.1589	5.21
